# Are quit attempts among U.S. female nurses who smoke different from female smokers in the general population? An analysis of the 2006/2007 tobacco use supplement to the current population survey

**DOI:** 10.1186/1472-6874-12-4

**Published:** 2012-03-19

**Authors:** Linda Sarna, Stella Aguinaga Bialous, Karabi Nandy, Qing Yang

**Affiliations:** 1 School of Nursing, University of California, Los Angeles, 700 Tiverton Ave, Los Angeles, CA 90095, USA; 2 Tobacco Policy International, San Francisco, CA 94118, USA

## Abstract

**Background:**

Smoking is a significant women's health issue. Examining smoking behaviors among occupational groups with a high prevalence of women may reveal the culture of smoking behavior and quit efforts of female smokers. The purpose of this study was to examine how smoking and quitting characteristics (i.e., ever and recent quit attempts) among females in the occupation of nursing are similar or different to those of women in the general population.

**Methods:**

Cross-sectional data from the Tobacco Use Supplement of the Current Population Survey 2006/2007 were used to compare smoking behaviors of nurses (n = 2, 566) to those of non-healthcare professional women (n = 93, 717). Smoking characteristics included years of smoking, number of cigarettes, and time to first cigarette with smoking within the first 30 minutes as an indicator of nicotine dependence. Logistic regression models using replicate weights were used to determine correlates of ever and previous 12 months quit attempts.

**Results:**

Nurses had a lower smoking prevalence than other women (12.1% vs 16.6%, *p *< 0.0001); were more likely to have ever made a quit attempt (77% vs 68%, *p *= 0.0002); but not in the previous 12 months (42% vs 43%, *p *= 0.77). Among those who ever made a quit attempt, nurses who smoked within 30 minutes of waking, were more likely to have made a quit attempt compared to other women (OR = 3.1, 95% CI: 1.9, 5.1). When considering quit attempts within the last 12 months, nurses whose first cigarette was after 30 minutes of waking were less likely to have made a quit attempt compared to other females (OR = 0.69, 95% CI: 0.49, 0.98). There were no other significant differences in ever/recent quitting.

**Conclusions:**

Smoking prevalence among female nurses was lower than among women who were not in healthcare occupations, as expected. The lack of difference in recent quit efforts among female nurses as compared to other female smokers has not been previously reported. The link between lower level of nicotine dependence, as reflected by the longer time to first cigarette, and lower quit attempts among nurses needs further exploration.

## Background

Smoking is a significant concern for women's health [1,2] including the health of the predominantly female nursing profession [3]. Viewing smoking among women by professional group provides a context for understanding the culture of smoking behavior and supporting targeted quit efforts. The purposes of this analysis of the Tobacco Use Supplement-Current Population Survey (TUS-CPS) are to explore smoking and quitting characteristics among female nurses as compared to women in the general population, and to examine factors associated with quit attempts which might support tailored smoking cessation programs for a predominantly female health profession.

In 1964 a Surgeon General Report demonstrated the negative impact of smoking on health, using primarily data from men [4]. The report had no impact on decreasing smoking among women. Subsequent Surgeon General Reports on smoking and women's health were published in 1980 [5] and 2001 [2], using extensive data from the Nurses' Health Study [6]. Thus, female nurses significantly contributed to our understanding of the impact of smoking on women and the benefits of quitting [7]. Analysis of smoking trends among participants in the Nurses' Health Study revealed that quitting smoking continued to be difficult across age groups, with nurses who continued to smoke having decreased survival [8] and lower quality of life [9].

Over 46 million Americans 18 years and older currently smoke (17.9% of women and 23.5% of men) [10] with slower rates of decline among women, pointing to a need to better understand quitting behavior among women. Differences of smoking rates by ethnicity, education and income exist among women and parallel that of smokers in the general population, with highest prevalence among women with lower levels of education and income [10].

Smoking is highly addictive and only 3% to 5% of those making an unaided quit attempt are abstinent one year later [11]. In 2008, 45.3% of current adult smokers (20.8 million people) stopped smoking for one day or more in the previous year because they were trying to quit [12]. A report of quit attempts among women of reproductive age indicated that while the majority of daily smokers made a quit attempt, fewer than half were successful in quitting, and only one fourth of daily smokers in the 18-24 years of age group succeeded [13]. Similar to smoking, quitting is inversely associated with education level [10].

Differences in smoking among occupational groups also exist which support the need for easily accessible tobacco dependence treatment appropriate to the characteristics of the workers and the worksite. Among occupational groups, current smoking among healthcare practitioners, reported at 11.8%, was not separated by sex [14]. Understanding smoking prevalence by occupation, especially in largely female occupations, is useful for developing strategies to support quit efforts of female smokers. Comparison of smoking behaviors of female nurses with other women provides a new perspective on the culture of smoking and quitting behaviors in the workplace. Changes in smoking among healthcare providers have been reported (1974-1991) with smoking declining more rapidly among physicians than among Registered Nurses (RNs) and Licensed Practical Nurses [15]. An analysis of smoking among healthcare professionals in the 2003 and 2006/2007 TUS-CPS revealed that smoking among RNs was 10.7% and among LPNs, 20.6%, with physicians and dentists having clearly lower smoking prevalence (2.3% and 3.0%, respectively) [16]. There was no statistically significant change in current smoking between the 2003 and 2006/07 RN and LPN cohorts. Calculation of quit ratios, determined by the ratio of former smokers to ever smokers, indicated that LPNs were the only group with a lower quit ratio than the general public. Supporting the quit efforts of healthcare providers is vital because healthcare professionals who smoke may be less likely to assist patients who smoke to quit [17]. Although there have been national efforts to support nurse in their quit efforts [18,19], data about nurses' quitting are limited.

The primary research objectives for this study comparing female nurses who smoke to female smokers in the general population were to: 1) assess lifetime quit attempts and quit attempts within the previous 12 months, 2) evaluate smoking characteristics (i.e., age of initiation, years of smoking, number of cigarettes per day, and time to first cigarette), and 3) to determine the relationship of smoking characteristics to quit attempts.

## Methods

### Sample

The CPS, a federally-funded household survey, is conducted by the United States (U.S.) Bureau of Labor Statistics and is intended to be nationally representative; 70% respond by telephone [20]. A random sample is selected from occupied housing units using monthly multistage probability samples. The TUS has accompanied the CPS survey since 1992-93 [21]. Smoking status is self-reported and defined as follows: current smoker (smoked at least 100 cigarettes lifetime and responded "every day or some day" to "Do you **now **smoke cigarettes every day, some days, or not at all"), former smoker (smoked at least 100 cigarettes lifetime and responded "not at all" to the previous question), and never smoker (smoked fewer than 100 cigarettes lifetime). Infrequent or "sometime" smokers are defined as smokers who smoked less than 12 days per month. Frequent smokers are defined as smokers who smoked every day or greater than 12 days per month. Both groups are included in this analysis focused on quit attempts.

First, we identified all women in the 2006/2007 TUS-CPS. Data from female nurses (RNs, n = 2, 107 and LPNs, n = 459, n = 2, 566 total) were then identified by occupational code [21]. Using occupational codes, 37 women who were non-nurse healthcare professionals (i.e., physicians, physician assistants, pharmacists, dentists, respiratory therapists) were removed from the general female sample, resulting in a sample of women in the general population (n = 93, 717) who were not nurses or other healthcare professionals. Finally, we extracted data from all female current smokers, 18 years and older, resulting in 333 nurses and 15, 998 women in the general population who were smokers.

### Smoking characteristics and level of nicotine dependence

Smoking status categories included never, former and current smoker. Characteristics of smoking included age of initiation and years of smoking asked of all "ever" smokers (i.e., current and former). Age of initiation (age of regular smoking in this dataset) was dichotomized as ≤ 15 years and > 15 years of age, similar to the strategy by Messer [22]. In parallel with other analyses of the TUS-CPS, number of years of smoking were put in four categories (≤ 10, 11- 20, 21-30, 30+ years) [23,24]. Similarly, number of cigarettes per day was put in three categories (≤ 10, 11-20, and > 20 cigarettes per day) as in other TUS-CPS analyses [25,26]. As a simple screener for level of nicotine dependence, we used the reported time to first cigarette (TTFC) after waking which has been identified as a strong predictor of successful quitting in previous analyses of the TUS-CPS [25,26]. Lower level of nicotine dependence was indicated by smoking *after *30 minutes of waking and higher level of dependence was indicated by smoking *within *30 minutes of waking.

### Quit attempts

The primary outcome for this analysis was quit attempts: ever trying to quit smoking and trying to quit within the prior 12 months. History of quit attempts was assessed by responses in the TUS-CPS through one of the following questions: "Have you ever stopped smoking for one day or longer because you were trying to quit smoking?" or "Have you ever made a serious attempt to stop smoking because you were trying to quit, even if you stopped for less than a day?". As demonstrated by Hughes & Callas [27], the inclusion of all quit attempts, including those that last less than 24 hours is important to eliminate bias towards underestimating quitting efforts. Thus, we also included quit attempts that lasted less than 24 hours.

### Demographic characteristics

Demographic characteristics examined among current smokers in the two groups included: age, race, education, marital status, income, and geographic location. To facilitate comparisons and to allow us to compare our findings with other studies using the TUS-CPS (22-24), we created several categorical variables. Age was grouped into four categories (18 - 24, 25 -44, 45 - 59, 60+ years), race was dichotomized (White/Non-White), education was dichotomized (< Bachelor's degree, Bachelor's degree or more), marital status was collapsed into three categories (single, married, widowed/separated/divorced). According to the original data categories, income is reported in three levels (< $19, 999, $20, 000-39, 999, $40, 000+) and state was collapsed into specific regions (Northeast, Midwest, South, and West).

For current smokers who were currently employed, we compared female nurses and women in the general population on support for quitting in the worksite through responses to: "Within the past 12 months, has your employer offered any stop smoking program or any other help to employees who want to quit smoking?". For current smokers who had visited a doctor in the previous 12 months, we compared the two groups for doctor's advice to quit. For smokers who made a quit attempt in the prior 12 months, we compared use of a telephone quitline for support.

### Data source and statistical analyses

The TUS-CPS data and questionnaire are available for free download from the National Cancer Institute at http://riskfactor.cancer.gov/studies/tus-cps/info.html. Statistical analyses were performed using SAS software (Cary, NC) version 9.1 and SAS-Callable SUDAAN version 9.0.1 (RTI International, Research Triangle Park, NC). Calculations of standard errors were based on the U.S. Census Bureau replicate weights, obtained from the National Cancer Institute and SUDAAN, which adjusts for complex survey design. Estimates of prevalence were calculated using self-response weights.

Differences in the distributions of female nurses and other females in the general population across levels of different categorical demographics variables and smoking characteristics were assessed using chi-square tests. This univariate analysis was done to identify potential factors that may have some effect on the outcomes in later model building of quit attempts.

Differences in ever quit attempts (any versus none) in lifetime as well as quit attempts in the previous 12 months between the two groups also were assessed using chi-square tests. The latter was done as a first step towards comparing the outcomes between the two groups without accounting for confounders. Then, to account for potential confounders, we built weighted logistic regression models for the outcomes separately using replicate weights. For each outcome, an initial model was built that included all the significant variables obtained from univariate analyses as well as others that were considered meaningful towards prediction of the outcome. Main effect of group as well as interaction terms involving group with other variables were entered into this initial model. The purpose of including interaction terms was to see if a variable had differential effects on the outcomes by varying group. At this stage, the model was assessed for goodness-of-fit and redundant variables (i.e., made no significant contribution to the prediction) were discarded and this process was repeated iteratively until a final model was obtained where all remaining variables contributed significantly to the prediction of each ever and recent quit attempts and showed good model fit. Both the final models included interaction terms involving group so we looked at the corresponding simple effects and presented these as the final results from the models. Goodness-of-fit of the models were assessed using Hosmer-Lemeshow test as well as studying the area under the curve (AUC) of the associated receiver operating characteristic (ROC) curves.

## Results

In the TUS-CPS 2006/2007, there were significant differences in smoking status between female nurses and women in the general population as displayed in Table 1, with a higher proportion of current smokers in the general population. In terms of any quit attempts (lifetime or within the previous 12 months), nurses were significantly more likely to have ever made a quit attempt, compared to other women (*p *= 0.0002). However, there were no significant differences between the two groups in their quit attempts in the previous 12 months (*p *= 0.77). Fewer nurse smokers who ever made a quit attempt, quit for less than 24 hours as compared to women in the general population (3.3% vs 5.2%); similarly, fewer nurses who made a quit attempt in the previous 12 months quit for less than 24 hours (10.5% vs 13.0%), but neither of these differences was statistically significant.

**Table 1:** Comparison between female nurses and females in the general population in smoking and quitting attempts

		Female nurses	Females in general population	p-value
		**(N = 2566, LPNS = 459; RNs = 2107)**	**(N = 93717)**	
	
		**n** (%, SE*)	**n** **(%, SE)**	

**Smoking Status**				
**Never Smokers**		1751 (71.1, 1.0)	59513 (66.1, 0.2)	< 0.0001
**Former Smokers**		482 (16.8, 0.8)	18206 (17.2, 0.2)	
**Current Smokers**		333 (12.1, 0.7)	15998 (16.6, 0.2)	

**Quit attempts among current smokers**				
**Ever made a quit attempt?**	Yes	256 (76.6, 2.6)	11085 (67.6, 0.5)	0.0002
	No	77 (23.4, 2.6)	4913 (32.44, 0.5)	
**Quit attempt in previous 12 months**	Yes	145 (43.2, 3.0)	6829 (42.3, 0.5)	0.77
	No	188 (56.8, 3.0)	9169 (57.7, 0.5)	

SE = standard error

Table 2 compares the demographic and smoking characteristics of current smokers in both groups. There were statistically significant differences in age, race, marital status, education and income, with a higher proportion of women having smoked for 10 years or less compared to nurses. A higher proportion of women smoked more than a pack a day when compared with female nurses.

**Table 2:** Comparison of female nurses who smoke and other female smokers in demographic and smoking characteristics

Variables	Female Nurses (n = 333)	Females in the general population (n = 15, 998)	p - value
**Demographic Characteristics**	**n** **% (SE)**	**n** **% (SE)**	

Age			< 0.0001
18-24	9 (3.2, 1.3)	1, 588 (14.5, 0.3)	
25-44	164 (48.4, 3.3)	6, 654 (41.1, 0.4)	
45-59	139 (41.6, 3.1)	5, 159 (30.7, 0.4)	
60 +	21 (6.9, 1.6)	2, 561 (13.7, 0.3)	

Race			
White	290 (84.7, 2.6)	12, 957 (78.2, 0.4)	0.01
Non-White	43 (15.3, 2.6)	3041 (21.8, 0.4)	

Education			
Less than Bachelor's Degree	235 (69.4, 2.8)	14, 105 (88.4, 0.3)	< 0.0001
Bachelor's degree or more	98 (30.6, 2.8)	1893 (11.6, 0.3)	

Marital status			
Married	163 (49.0, 3.3)	6, 937 (41.7, 0.5)	0.0005
Widowed, divorced, separated	112 (34.1, 3.0)	5, 460 (32.1, 0.5)	
Never married	58 (16.9, 2.3)	3601 (26.2, 0.4)	

Region			
Northeast	72 (20.8, 3.2)	2, 966 (17.4, 0.4)	0.48
Midwest	100 (28.8, 2.9)	4, 380 (26.6, 0.4)	
South	97 (34.8, 3.2)	5, 339 (38.5, 0.5)	
West	64 (15.7, 2.3)	3, 313 (17.6, 0.4)	

Income			
Less than or equal to $19, 999	18 (6.4, 1.7)	4, 318 (27.1, 0.4)	< 0.0001
$20, 000 - $39, 999	63 (20.6, 2.5)	4, 204 (26.0, 0.5)	
$40, 000+	252 (73.0, 2.8)	7, 476 (47.0, 0.5)	

**Smoking Characteristics**	**N** **% (SE)**	**n** **% (SE)**	

Age of smoking initiation of regular smoking			
Less than or equal to 15 years of age	78 (24.5, 3.0)	4, 800 (30.3, 0.4)	0.06
Greater than 15 years of age	255 (75.5, 3.0)	11, 198 (69.7, 0.4)	

Years of smoking			
Less than or equal to 10 years	109 (33.1, 3.1)	5, 643 (40.0, 0.4)	0.005
11 - 20 years	88 (25.1, 2.8)	3, 485 (21.3, 0.3)	
21 - 30 years	81 (25.6, 3.0)	3, 213 (18.7, 0.3)	
Greater than 30 years	55 (16.2, 2.3)	3, 657 (20.2, 0.3)	

Number of cigarettes per day			
≤ 10	190 (56.3, 2.8)	8, 370 (53.9, 0.5)	0.0001
11 - 20	130 (40.3, 2.9)	6, 288 (38.3, 0.5)	
> 20	13 (3.3, 1.0)	1, 340 (7.8, 0.3)	

1st cigarette within 30 minutes of waking up			
Yes	159 (52.1, 3.2)	8, 368 (52.0, 0.5)	0.97
No	171 (47.9, 3.2)	7, 362 (48.1, 0.5)	

Worksite support for quitting smoking was evaluated among those who were not retired, had been working for pay and are not self-employed. Female nurses reported significantly higher worksite support for quitting compared to females in the general population (42.0% vs. 17.1%, *p *< 0.0001). Among those who had seen a medical doctor in the past 12 months, there was no significant difference in the frequency of a doctor's advice to quit smoking. For those who made a quit attempt in the prior 12 months, only a small proportion in both groups reported using a telephone quitline to support quit efforts, with a significantly lower proportion of nurses reported using a quitline when compared to other women (0.6% vs. 3.4%, p < 0.0001).

Table 3 displays the logistic regression models for each of the outcomes: ever quit attempts (versus none) and any quit attempts in the previous 12 months for nurses and women in the general population, controlling for statistically significant group differences in demographic variables and smoking characteristics, as observed in Table 1.

**Table 3:** Multiple logistic regression models for female smokers' *ever *and previous 12 month quit smoking attempts

Interaction between occupation category and time to first cigarette within 30 min of waking up	Adjusted OR	95% CI	p-value
Model I: **Ever **made a quit attempt^a^			

• Female nurses v. other women who smoke WITHIN 30 mins of waking up	3.1	(1.9, 5.1**)**	< 0.0001
• Female nurses v. other women who smoke AFTER 30 mins of waking up	1.1	(0.7, 1.6)	0.66

Model II: Made a quit attempt in the previous 12 months^b^			

• Female nurses v. other women who smoke WITHIN 30 mins of waking up	1.3	(0.9, 1.9)	0.11
• Female nurses v. other women who smoke AFTER 30 mins of waking up	0.69	(0.49, 0.98)	0.04

^a ^Controlling for demographics (age, race, marital status) and smoking characteristics (age of smoking initiation, smoking 12 or more days a month)

^b ^Controlling for demographics (region) and smoking characteristics (years of smoking, cigarettes per day, age of smoking initiation, smoking 12 or more days a month)

In the model for ever making a quit attempt, there was a significant (*p *= 0.0012) interaction between group and TTFC (as an indicator of high/low nicotine dependence), controlling for age, race, marital status, smoking for more than 12 days in a month, and age of smoking initiation of regular smoking. When there is a significant interaction term, it is customary to look at the simple effects i.e., fix the level of one variable and test for differences among the levels of the other variable and repeat this for both variables. In this analysis of female smokers, we tested for differences between nurses and other women. Thus, looking at the simple effect of TTFC, we find in Table 3 (Model I) that after controlling for confounders, among those with a high level of nicotine dependence, female nurses had three times the odds of having ever made a quit attempt compared to that of other women in the general population (OR = 3.1, 95% CI: 1.9, 5.1). Among those with low level of nicotine dependence, there was no significant difference in lifetime quit attempts between the two groups. The AUC of the associated ROC curve for this model was 0.6 and the p-value from the Hosmer-Lemeshow test was 0.18, indicative of good model fit.

In the model for quit attempts in the previous 12 months, there was a similar significant interaction between group and TTFC (*p *= 0.0111), controlling for geographic region, smoking for more than 12 days in a month, years of smoking, cigarettes per day and age of smoking initiation. Looking at the simple effect of TTFC, we find in Table 3 (Model II) that after controlling for confounders, among those with a low level of nicotine dependence, nurses were less likely to have made a quit attempt in the previous 12 months compared to other females during that same time (OR = 0.69, 95% CI: 0.49, 0.98). However, among those with high level of nicotine dependence, there were no significant differences between the two groups in quit efforts in the previous 12 months. AUC of the associated ROC curve from the final fitted model was 0.6, implying good fit. This was further confirmed by the Hosmer-Lemeshow test, *p*-value of 0.71.

## Discussion

This analysis revealed that although having lower smoking prevalence than women in the general population, female nurses who smoke may not have an easier time quitting and one should, therefore, not assume, that these healthcare professionals do not need support in quitting. Over three quarters of the nurses who were current smokers had made a quit attempt, higher than the general female population, but less than half had made an attempt within the previous 12 months. The overall prevalence of quit attempts by nurse smokers (76.6%) was higher than previously reported. In a 27-year follow-up (1976-2003) of participants in the Nurses' Health Study, 69% of the nurse who smoked had never made a quit attempt [8]. In the TUS-CPS sample of female smokers, the proportions of nurses and women making a quit attempt in the previous 12 months (43% and 42%, respectively) are slightly lower than the one reported for the general population, i.e., 45% [28]. The lack of difference in recent quit efforts also was surprising given the changes in social acceptability of smoking, especially among healthcare professionals [29]. This is substantially lower than the quit attempt rates of a motivated sample of nurse smokers (92% female) participating in web-based smoking cessation intervention where 73% had made a quit attempt in the previous 12 months [19].

The difference in the demographics (i.e., age, race, education, marital status, income) between nurses who smoke compared to other female smokers in the TUS-CPS sample has not been previously described. It was expected that nurses would be in the mid-range age groups, and that they would have a higher proportion of Whites and level of income as this mirrors the demographics of the profession [30,31], and is similar to another sample of nurse smokers [19].

Female nurses who smoked also differed from other women in the TUS-CPS in smoking characteristics. Nurses had more years of smoking but because of the older age of this group, differences with the general female population would be expected. The categories of number of cigarettes smoked per day also were significantly different with a higher proportion of nurses in the low category (fewer than 10 cigarettes per day) and in the mid-range (11 - 20 cigarettes per day) and fewer smoking more than 20 cigarettes per day. This is similar to the 67% of nurse smokers participating in a web-based smoking cessation program who smoked less than 20 cigarettes per day [19,32]. A trend toward fewer cigarettes also was noted in 1986 in the Nurse's Health Study, with highest number of cigarettes smoked among the cohort born in 1940-44 [6]. There was no significant difference in the TTFC. More than half of nurses and women in the general population fall into the higher level of nicotine dependence category of smoking within 30 minutes of awakening, an indicator of addiction and of less successful quit efforts [25]. This is similar to a previous report of nurse smokers [19].

### Support for quitting

Nurses have acknowledged that smoking can disrupt workplace relationships and that support for quitting in the workplace would enable quit attempts [19,33,34]. In this sample, nurses reported significantly higher levels of worksite cessation support than other women. There were no significant differences in advice from physicians to quit with over a third of nurses and other women not receiving such advice. The proportion of those receiving advice to quit from a healthcare provider was similar to other reports of quit efforts among nurses [19] and among smokers in general [35].

In this version of the TUS-CPS there are limited questions about cessation support. We do not have information about use of medications or counseling, the evidence-based recommended strategies for improving quit rates [11]. In a previous report of nurses' quit efforts, a minority of nurses utilized pharmacotherapy and counseling to help them quit smoking [19]. In focus groups of current and former smokers, nurses exhibited similar misconceptions about pharmacotherapy as the general population with reluctance to "replace one addiction with another" and a preference for quitting on their own [33]. Thus, despite being healthcare professionals, nurses who smoke may not be aware of evidence-based methods for quitting. The fact that only a small proportion of the sample ever used a telephone quitline for cessation support emphasizes the need to promote this free and effective resource more broadly. Few smokers seem to take advantage of this resource [36], including nurses making a quit attempt [19]. A campaign promoting the quitline for nurses and for other female smokers might be warranted. Nurses could take advantage of this private, individualized cessation support.

### Correlates of quitting

While smoking the first cigarette within 30 minutes of waking up was not a predictor for nurses making at least one quit attempt in the previous 12 months, it was a predictor for nurses making at least one quit attempt in their lifetime. Compared to other female smokers, nurses who were had lower levels of nicotine dependence (i.e., longer TTFC) were less likely to have made a quit attempt in the previous 12 months. This has not been previously reported. If nurse smokers, as compared to other female smokers, experience fewer symptoms of withdrawal and addiction, could this lead them to minimize health risks of smoking and fewer quit attempts? This clearly is an area needing further study. There are a variety of barriers to quitting reported by nurses. Some nurses who smoke have acknowledged their fear of losing work break if they quit [19,37] and there are reports of nurses who smoke getting more work breaks [35]. Nurses also have described difficulties in coping with withdrawal symptoms during the work shift and concerns of losing friendships of "smoking buddies" [35] which may be a concern for other female smokers as well.

Unlike other female professions, the negative impact of smoking on the health of nurses has been well established [8]. The trend in decline of smoking among nurses in the past several decades mirrors changes in smoking among women. However, this study reveals some differences in quitting behaviors that are of concern and points to the need for ongoing programs encouraging nurses to make quit attempts and utilize available resources, such as the telephone quitline. Further, given that smoking among nurses remains a barrier to the provision of smoking cessation intervention to patients, all efforts should be made to promote cessation resources for nurses' own use. Evidence suggests that worksite smoking bans may encourage further quit attempts [38]. As more and more medical campuses become smokefree, this may accelerate quit efforts of all healthcare professionals [39,40].

### Limitations

When using the TUS-CPS database, there are a number of limitations that must be considered in the interpretation of these findings. Although these are weighted estimates, the sample size of nurses is relatively small. By merging data of RNs and LPNs, this may have resulted in a cohort with higher smoking prevalence and lower education than the RN population alone, but the sample of LPNs was too small to be examined separately. In a related analysis of male and female healthcare providers, LPNs had the highest smoking prevalence (20.6%) and the lowest quit ratio [29]. Thus, we are unable to provide specific directions for either group individually. This analysis focuses on female smokers, however smoking prevalence among male healthcare providers also is a concern. Additionally, we were limited in sub-set analysis because of the sample size.

Smoking status is self-reported and not biochemically confirmed, as recommended [41]. As described, the 2006/2007 version of the TUS-CPS is limited in the number of questions about quitting. Thus, we are unable to describe the range of resources used by women to quit and other variables which might impact their quit such as smoking among household members. No information is available on number of quit attempts although multiple attempts is common [32,33]. This dataset does not provide information about triggers for quit attempts. Nurses have reported pregnancy, health concerns and illness among family members as incentives to quitting as well as the cost of tobacco products, smokefree work environments, and the social unacceptability of smoking [32]. Additionally, we do not have information on body weight. Being overweight and weight gain after quitting have been suggested as barriers to quit efforts [2].

## Conclusions

This analysis provides information on similarities and differences in quit attempts among female nurses who smoke and female smokers in the general population. Although the relatively low prevalence of smoking among nurses is encouraging, it remains much higher than the estimated 2% of physician smokers [29]. Not previously reported, there was no difference in recent quit efforts among nurse smokers compared to other women. Also of interest was the finding that among those who made a recent quit attempt, nurses with a lower level of nicotine dependence were less likely to try to quit than other female smokers in this group. Different occupational groups may have different acceptability in the culture of smoking and quitting. Research exploring smoking and quitting in predominantly female occupations may be important in describing the culture of smoking and quitting and finding ways to address this major health risk affecting women.

## Competing interests

The authors declare that they have no competing interests.

## Authors' contributions

LS and SAB conceived of the study; KN contributed to the study design and devised the analysis plan and data display with the assistance of QY. All authors participated in the interpretation of the data and writing and review of the manuscript. LS and SAB obtained funding to partially support the project. All authors read and approved the final manuscript.

## Pre-publication history

The pre-publication history for this paper can be accessed here:

http://www.biomedcentral.com/1472-6874/12/4/prepub
